# High-Accuracy 3D Gaze Estimation with Efficient Recalibration for Head-Mounted Gaze Tracking Systems

**DOI:** 10.3390/s22124357

**Published:** 2022-06-08

**Authors:** Yang Xia, Jiejunyi Liang, Quanlin Li, Peiyang Xin, Ning Zhang

**Affiliations:** 1 State Key Laboratory of Digital Manufacturing Equipment and Technology, School of Mechanical Science and Engineering, Huazhong University of Science and Technology, Wuhan 430074, China; xiayang_2179@163.com (Y.X.); liquanlin@hust.edu.cn (Q.L.); py.xiiin@gmail.com (P.X.); 2 National Research Center for Rehabilitation Technical Aids, Beijing 100176, China; zhangning@nrcrta.cn

**Keywords:** head-mounted gaze tracker, visual axis, 3D gaze estimation, head pose tracking, recalibration, polynomial regression

## Abstract

The problem of 3D gaze estimation can be viewed as inferring the visual axes from eye images. It remains a challenge especially for the head-mounted gaze tracker (HMGT) with a simple camera setup due to the complexity of the human visual system. Although the mainstream regression-based methods could establish the mapping relationship between eye image features and the gaze point to calculate the visual axes, it may lead to inadequate fitting performance and appreciable extrapolation errors. Moreover, regression-based methods suffer from a degraded user experience because of the increased burden in recalibration procedures when slippage occurs between HMGT and head. To address these issues, a high-accuracy 3D gaze estimation method along with an efficient recalibration approach is proposed with head pose tracking in this paper. The two key parameters, eyeball center and camera optical center, are estimated in head frame with geometry-based method, so that a mapping relationship between two direction features is proposed to calculate the direction of the visual axis. As the direction features are formulated with the accurately estimated parameters, the complexity of mapping relationship could be reduced and a better fitting performance can be achieved. To prevent the noticeable extrapolation errors, direction features with uniform angular intervals for fitting the mapping are retrieved over human’s field of view. Additionally, an efficient single-point recalibration method is proposed with an updated eyeball coordinate system, which reduces the burden of calibration procedures significantly. Our experiment results show that the calibration and recalibration methods could improve the gaze estimation accuracy by 35 percent (from a mean error of 2.00 degrees to 1.31 degrees) and 30 percent (from a mean error of 2.00 degrees to 1.41 degrees), respectively, compared with the state-of-the-art methods.

## 1. Introduction

As an effective way of revealing human intentions, gaze tracking technology has been widely applied in many areas, including marketing, ergonomics, rehabilitation robots and virtual reality [1,2]. Gaze tracking systems can be divided into remote and head-mounted gaze trackers (HMGT) [3]. The remote gaze tracker is typically placed on a fixed location such as a desktop, to capture images of the user’s eyes and face by a camera. The HMGT system is usually fixed to the user’s head, which includes the scene camera to capture the view of the scene and eye camera to observe eye movement. The feature of allowing users to move freely makes HMGT more flexible and suitable for tasks such as human–computer interaction in a real 3D environment. Therefore, HMGT has received extensive attention by many researchers in recent years.

The problem of 3D gaze estimation can be viewed as inferring visual axes from eye images captured by cameras. Typically, there are two different gaze estimation methods which are model-based and regression-based methods, respectively. The model-based methods utilize extracted features from eye images to build a geometric eye model and calculate the visual axis. Traditional model-based methods employed multiple eye cameras and infrared light sources to calculate optical axis, and then calculate the angle Kappa between the optical axis and the visual axis with single-point calibration [4,5]. The main merits are rapid calibration and robustness against system drift (the slippage of HMGT), but the complex setting of cameras and lights requirements limit its application. As the HMGT with simple camera setting can be developed more conveniently, it has a broader application prospect. Some research utilized inverse projection law to calculate the pupil pose with simple camera setting. The contour-based method in [6] designs a 3D eye model fitting method to compute a unique solution by fitting a set of eye images, but the gaze estimation accuracy is relatively low due to the corneal refraction of the pupil. The method in [7] models the corneal refraction by assuming the physiological parameters of the eyeball, but the performance is not stable because physiological parameters of the eyeball vary from person to person. In addition, it is challenging for these methods to extract the pupil’s contour accurately in the eye image due to the occlusion of eyelids and eyelashes. Therefore, it is difficult for model-based methods to get high-accuracy visual axes with a simple camera setting.

In contrast, the regression-based methods usually adopt single eye camera. The key idea of this kind of method is to establish a regression model to fit the mapping relationship between eye image features and gaze points in scene camera coordinate system [8,9]. This kind of method has two sources of error, namely parallax error and extrapolation error. The noticeable extrapolation error may occur due to the underfitting situation caused by improper regression models or calibration point sampling strategy. The parallax error is caused by the spatial displacement between the eyeball and the scene camera [10]. For instance, the corresponding eye image features of the points on visual axis are the same, but their coordinates in the scene camera coordinate system are different, which leads to one-to-many relationships.

To reduce extrapolation error, different mapping functions are investigated, in which the polynomial regression is the most common model. The method in [11] compares different polynomial functions and chooses the best performer to estimate the gaze point. However, the functions higher than two orders can not reduce extrapolation errors significantly [12]. In [1,13], the Gaussian process regression is investigated as an alternative mapping function, but the accuracy performance of the Gaussian process regression is unstable. To improve the estimation accuracy of gaze depth, some methods employ MLP neural network to estimate the depth with inputs of pupil centers or pupillary distance [14,15], but the gaze estimation models based on neural network require more training data, which causes a heavier burden of calibration procedures.

To prevent parallax error, some methods determine the depth of 3D gaze point by analyzing scene information. The method in [16] uses SLAM to extract environmental information. Then, the 3D gaze point is estimated by using the correspondence relationship between the triangles containing 2D gaze points in the scene camera image and triangles containing 3D gaze points in the real world. In [17], SFM (Structure from Motion) is utilized to estimate the 3D gaze point, with two different head positions to look at the same place. However, the performance of these methods gets worse when acquiring sparse feature points from scene image. A more common method is to calculate the visual axes of both eyes and intersect them to get 3D gaze point. The method in [18] sets calibration points on a screen with fixed depth and requires the user to keep the head still, then employs a polynomial function to fit the mapping relationship between 2D pupil center and 3D gaze point. The visual axis is determined by the fixed eyeball center and the estimated gaze point on the screen. As an improved method, the method in [19] requires two additional calibration points outside the mapping surface, then a more precise position of the eyeball center is calculated by triangulation. In [9], the calibration data are collected by staring at a fixed point while rotating head, the position of the eyeball center is set to an estimated initial value, and the loss function based on the angular error of the visual axis is employed to optimize parameters. Obviously, the above methods infer the visual axis by calculating the eyeball center and the direction of line of sight. However, the eyeball centers are usually estimated with data-fitting methods, which can be sample dependent and have limited generalization ability.

In summary, existing regression-based paradigms face three main issues. The first one is how to formulate an appropriate regression model. Most paradigms utilize the image pupil center and the gaze point as input and output features [9,19]. However, it may lead to inadequate fitting performance and appreciable extrapolation errors due to the complexity of the human visual system. The second one is how to define a proper calibration point distribution over the whole field of view. Existing paradigms sample the calibration points over a casual field of view [9,20]. However, a significant accuracy degradation would occur when the gaze direction is outside the calibration range due to the extrapolation error. The third one is the lack of an elegant recalibration strategy. The mapping relationship between input and output features would change as the HMGT slips. Without an efficient recalibration strategy, the user needs to repeat primary calibration procedures to rectify relative parameters of the gaze estimation model with a heavy burden [21,22].

To address these issues, a hybrid gaze estimation method is proposed with real-time head pose tracking in this paper. On one hand, it utilizes the human eye geometric model to analyze the parameters that influence the pose of visual axis and estimates the key parameters eyeball center and camera optical center in head frame. On the other hand, it employs a polynomial regression model to calculate the direction vector of the visual axis. The main contributions of this paper are summarized as follows:

(1) A novel hybrid 3D gaze estimation method is proposed to achieve higher gaze estimation accuracy than the state-of-the-art methods. The two key parameters, eyeball center and camera optical center, are estimated in head frame with geometry-based method, so that a mapping relationship between two direction features is established to calculate the direction of the visual axis. As the direction features are formulated with the accurately estimated parameters, the complexity of mapping relationship is reduced and a better fitting performance can be achieved.
(2) A calibration point sampling strategy is proposed to improve the uniformity of training set for fitting the polynomial mapping and prevent appreciable extrapolation errors. By estimating the pose of the eyeball coordinate system, the calibration points are retrieved with uniform angular intervals over human’s field of view for symbol recognition.
(3) An efficient recalibration method is proposed to reduce the burden of recovering gaze estimation performance when slippage occurs. A rotation vector is introduced to our algorithm, and an iteration strategy is employed to find the optimal solution for the rotation vector and new regression parameters. With an updated eyeball coordinate system, only one extra recalibration point is enough for the algorithm to get comparable gaze estimation accuracy with primary calibration.

The rest of the paper is organized as follows. Section 2 describes the proposed methods in primary calibration and recalibration. Section 3 presents the experimental results. Section 4 is the discussion, and Section 5 is the conclusion.

## 2. Materials and Methods

### 2.1. Model Formulation

The key point of 3D gaze estimation is to estimate the visual axis in a scene camera coordinate system by analyzing images captured by the eye camera. To design a high-accuracy gaze estimation model to calculate the visual axis, the relationship between eye image features and visual axis is derived based on a geometric eye model [23].

**Figure 1:**
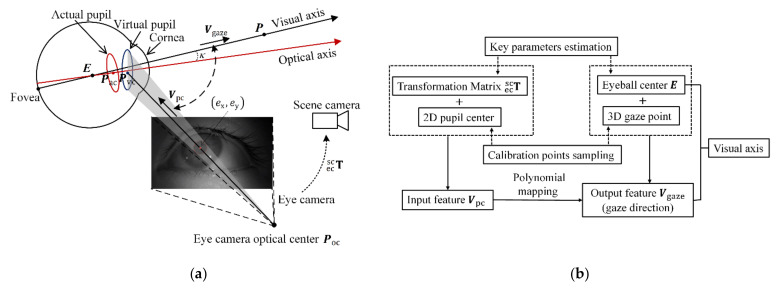
Illustration of gaze estimation model. (**a**) Each eye image feature (2D pupil center) corresponds to a vector $V_{\mathrm{pc}}$ which is used as the input feature to calculate the vector $V_{\mathrm{gaze}}$. Noted that E is assumed as the intersection of all visual axes. (**b**) Flowchart of the model formulation.

As shown in Figure 1a, the optical axis passes through the eyeball center $E\in {\mathbb{R}}^{3\times 1}$ and actual pupil center $P_{\mathrm{ac}}\in {\mathbb{R}}^{3\times 1}$. The visual axis is represented as the line formed by eyeball center E and gaze point $P\in {\mathbb{R}}^{3\times 1}$. There is an angle κ between the optical axis and visual axis. Because of the corneal refractive power, the pupil captured by the eye camera is not actual pupil but virtual pupil. The 2D pupil center $\left(e_{x},e_{y}\right)$ can be connected with eye camera optical center $P_{\mathrm{oc}}\in {\mathbb{R}}^{3\times 1}$ to form one straight line passing through $P_{\mathrm{vc}}\in {\mathbb{R}}^{3\times 1}$, whose direction vector is $V_{\mathrm{pc}}$. $V_{\mathrm{pc}}$ can be calculated by

(1) P01=Tecsc·Kcam−1exey11Vpc=P0−Poc‖P0−Poc‖

where Tecsc is the transformation matrix between scene camera and eye camera, $K_{\mathrm{cam}}$ is the intrinsic matrix of eye camera and $P_{0}$ is a point on the line formed by $P_{\mathrm{oc}}$ and $P_{\mathrm{vc}}$. Then, the direction vector of visual axis, $V_{\mathrm{gaze}}$ can be calculated by

(2) Pvc−Poc=γ·VpcPac1=Tvpap·Pvc1Vgaze=Roava·Pac−E‖Pac−E‖

where Tvpap is the transformation matrix between actual pupil and virtual pupil and Roava is the rotation matrix between the visual axis and optical axis. γ is the offset distance between eye camera optical center and virtual pupil center. Thus, the point P on the visual axis can be calculated by

(3) $$P=E+\lambda \cdot V_{\mathrm{gaze}}$$

where λ is a proportional coefficient. To calculate the visual axis, Tecsc,E,γ,Roava,Tvpap need to be estimated. The flowchart of the calculation is shown as Figure 1b. For the key parameters Tecsc and E, the accurate values are estimated with proposed geometry-based method. The details are described in Section 2.2 and Section 2.3. For other parameters γ, Roava and Tvpap that are related to corneal refraction, it is difficult to get accurate values. Because they are usually calculated with average eyeball physiological parameters which vary from person to person. By sampling calibration points, a quadratic polynomial model is employed to fit the nonlinear mapping from $V_{\mathrm{pc}}$ to $V_{\mathrm{gaze}}$, which actually reflects the inherent impacts of these parameters as shown in formula (2). The details are described in Section 2.4.

### 2.2. Estimation of the Transformation Matrix Tecsc

**Figure 2:**
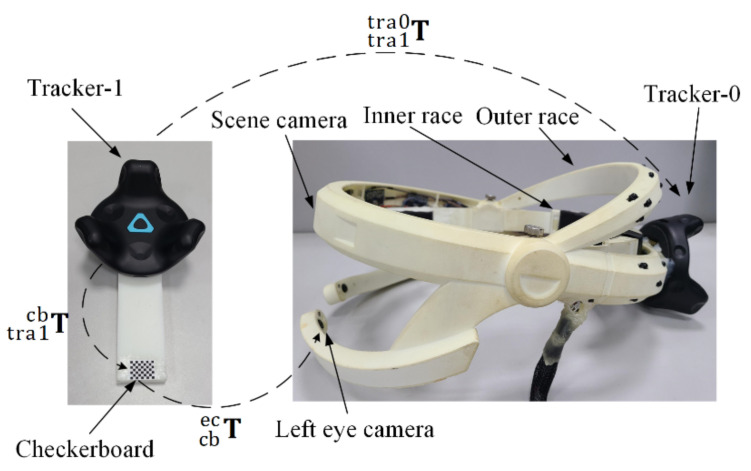
Illustration of estimation method for the cameras’ coordinate system. The left eye camera is taken for example.

The estimation method for calculating the transformation matrix between cameras and HMGT based on 6D pose trackers is shown in Figure 2. The left side is a calibration tool on which a checkerboard is fixed with a 6D pose tracker (Tracker-1). The right side is the developed HMGT. A 6D pose tracker (Tracker-0) is fixed with it to track the head pose. Noted that the transformation between different 6D pose trackers can be obtained in real time. During the calibration, we captured n images of checkerboard by the camera, saving the corresponding transformation matrix between Tracker-0 and Tracker-1 for each image frame. By utilizing the camera calibration toolbox in MATLAB, the transformation matrix Tcbec between the checkerboard and eye camera for each frame can be calculated. Then, the transformation matrix between Tracker-0 and the eye camera corresponding to the i’th images can be calculated by:

(4) Tectra0i=Ttra1tra0i·Ttra1cbi−1·Tcbec−1

The transformation matrix Tectra0i can be decomposed into a translation vector $t_{i}$ and a rotation matrix $R_{i}$. The rotation matrix $R_{i}$ can be converted to a quaternion $q_{i}$. To calculate the average transformation, the average translation vector $\bar{t}$ is calculated by

(5) $$\bar{t}=\frac{1}{n}\overset{n}{\underset{i=1}{\sum }}t_{i}$$

The average quaternion $\bar{q}$ is calculated by the proposed method in [24],

(6) $$\bar{q}=\arg\underset{q\in S^{3}}{\max}q^{T}Mq$$

where $S^{3}$ denotes the unit 3 sphere,

(7) $$M=\overset{n}{\underset{i=1}{\sum }}q_{i}q_{i}^{T}$$

The average quaternion $\bar{q}$ is the eigenvector of M corresponding to the maximum eigenvalue. Then, Tectra0 can be calculated by combining $\bar{q}$ and $\bar{t}$. Similarly, the transformation matrix Tsctra0 between the scene camera and Tracker-0 can be calculated. Then, the transformation matrix Tecsc between the scene camera and eye camera can be calculated by

(8) Tecsc=Tectra0·Tsctra0−1

As shown in Figure 2, cameras and Tracker-0 are fixed with the outer race, the inner race is fixed with the user’s head when the system is working, and the outer race can rotate relative to the inner race for suitable wearing. Therefore, the transformation between the cameras and Tracker-0 is constant, and the estimation for Tecsc is needed only once.

### 2.3. Estimation of the Eyeball Center E

**Figure 3:**
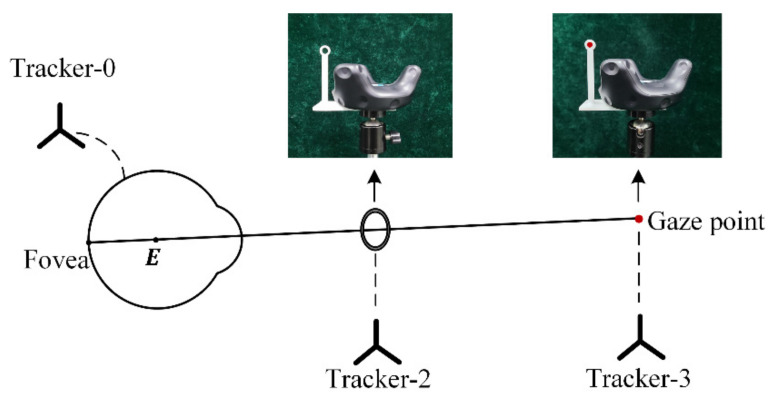
Estimation of the eyeball center E with developed calibration tools. The transformation between Tracker-2 and the small hole is predefined, and the coordinate of gaze point in Tracker-3 coordinate system is predefined.

Similar to most existing paradigms, the eyeball center ***E*** is assumed as the intersection of different visual axes. Figure 3 illustrates the estimation of the eyeball center by utilizing the developed calibration tools. The user is required to gaze at a point through a small hole with different head orientations, and the visual axis is regarded as the line formed by the gaze point and the center of the hole. Considering that the collected visual axes may be non-coplanar, the eyeball center E is calculated as the midpoint of the common perpendicular of two different visual axes. Although the eyeball center is estimated in the Tracker-0 coordinate system, it can be conveniently switched to the scene camera coordinate system with estimated Tsctra0 in the previous section.

**Figure 4:**
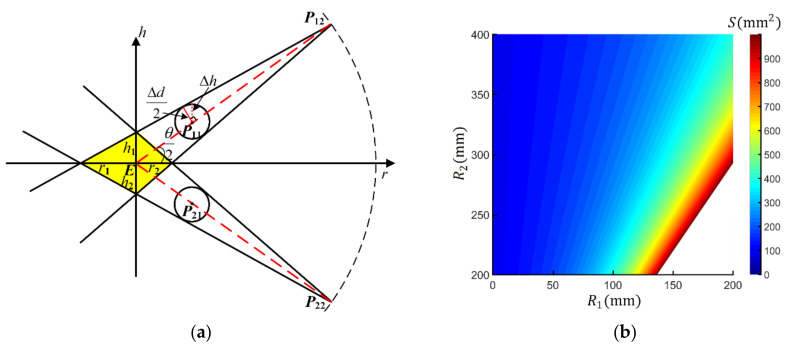
Error analysis of eyeball center estimation. (**a**) Error zone illustration. The distance between eyeball center and calibration tools are assumed as constant values. The *r*-axis refers to the line connecting E to the midpoint of $P_{12}P_{22}$. (**b**) The relationship between S and $R_{1}$, $R_{2}$ when θ=90°, Δd=10 mm. Note that the white region in the heat maps means the error is larger than $1000{\;\mathrm{mm}}^{2}$, and we do not show the detail for better observation.

The error analysis of eyeball center calibration is shown in Figure 4a. There are two different visual axes collected in the Tracker-0 coordinate system. The maximum error between the collected line and the visual axis is determined by the diameter ∆d of the hole. The two error cones formed by $P_{12}P_{22}$ and $P_{21}P_{22}$ intersect to form a yellow diamond-like error region on the r−h plane. Assuming that $R_{1}$ represents the distance between the eyeball center and the center of the small hole, $R_{2}$ represents the distance between the eyeball center and the gaze point. Based on triangular similarity, the region width $h_{1}$ satisfies,

(9) $$\frac{\Delta h}{h_{1}}=\frac{\left|\left|P_{11}P_{12}\right|\right|}{\left|\left|EP_{12}\right|\right|}=\frac{R_{2}-R_{1}}{R_{2}}$$

where

(10) $$\Delta h=\frac{\Delta d}{2\cos\left[\arccos\frac{\Delta d}{2(R_{2}-R_{1})}-\left(\frac{\pi }{2}-\frac{\theta }{2}\right)\right]}$$

As $\frac{\Delta d}{2(R_{2}-R_{1})}$ is small, $\arccos\frac{\Delta d}{2(R_{2}-R_{1})}\approx \frac{\pi }{2}$, then

(11) $$h_{1}=\frac{\Delta d}{2\left(1-\frac{R_{1}}{R_{2}}\right)\cos\frac{\theta }{2}}$$

Based on similar derivation, $h_{2}=h_{1},\;$the region depth $r_{1}$, $r_{2}$ satisfy

(12) $$r_{1}\approx r_{2}=\frac{\Delta d}{2\left(1-\frac{R_{1}}{R_{2}}\right)\sin\frac{\theta }{2}}$$

Then, the area S of error region on the r−h plane can be calculated by

(13) $$S=\frac{1}{2}\frac{\Delta^{2}d}{{\left(1-\frac{R_{1}}{R_{2}}\right)}^{2}\sin\frac{\theta }{2}\cos\frac{\theta }{2}}$$

Based on the above derivation, the strategies to improve the accuracy of eyeball center estimation can be concluded as:

(1) Reducing Δd, which is the inner diameter of the small hole;
(2) Reducing $\frac{R_{1}}{R_{2}}$, which means increasing the distance between the small hole and gaze point, and decreasing the distance between the small hole and the user (see Figure 4b);
(3) Setting the angle between two collected visual axes, θ=90°, considering the contradictory relation between region width and depth.

### 2.4. Regression Model Fitting

Utilizing estimated eyeball center E sc and Tecsc in the scene camera coordinate system, the set of input feature $V_{\mathrm{pc}}$ and output feature $V_{\mathrm{gaze}}$ can be obtained from training set. Then, a quadratic polynomial regression function is employed to fit the mapping relationship between $V_{\mathrm{pc}}$ and $V_{\mathrm{gaze}}$. Assuming $\;V_{\mathrm{pc}}={\left[x_{0},y_{0},z_{0}\right]}^{T}$,$\;V_{\mathrm{gaze}}={\left[x_{1},y_{1},z_{1}\right]}^{T}$, then

(14) $$V_{\mathrm{gaze}}=\left[\begin{array}{c} x_{1}\\ y_{1}\\ z_{1} \end{array}\right]∼\left[\begin{array}{c} \beta^{x}\psi \left(V_{\mathrm{pc}}\right)\\ \beta^{y}\psi \left(V_{\mathrm{pc}}\right)\\ \beta^{z}\psi \left(V_{\mathrm{pc}}\right) \end{array}\right]$$

where $\psi \left(V_{\mathrm{pc}}\right)={\left[x_{0}^{2},y_{0}^{2},z_{0}^{2},x_{0}y_{0},x_{0}z_{0},y_{0}z_{0},x_{0},y_{0},z_{0},1\right]}^{T}$. $\beta^{x}$, $\beta^{y}$, $\beta^{z}$ are 1×10 matrices. Assuming $\beta =\left[\beta^{x};\beta^{y};\beta^{z}\right],$ the aim is to calculate β by minimizing the average angle error between the estimated and real visual axis, which is given as

(15) $$\underset{\left\{\beta^{x},\beta^{y},\beta^{z}\right\}}{min}\frac{1}{N}\overset{N}{\underset{i=1}{\sum }}\arccos\left(\frac{\beta \cdot \psi \left(V_{\mathrm{pc}}^{i}\right)\cdot V_{\mathrm{gaze}}^{i}}{\left\|\beta \cdot \psi \left(V_{\mathrm{pc}}^{i}\right)\|\|V_{\mathrm{gaze}}^{i}\right\|}\right)$$

where *N* is the number of calibration points. For addressing the nonlinear optimization problem such as (15), it is paramount to initialize the parameters with reasonable values. Thus, the initial value of β is calculated by

(16) $$\beta_{\mathrm{init}}=T_{\mathrm{gaze}}\cdot \psi^{T}{\left(\psi \psi^{T}\right)}^{-1}$$

where ψ is the matrix holding $\left\{\psi \left(V_{\mathrm{pc}}^{i}\right)\right\}$ in the whole training set and $T_{\mathrm{gaze}}$ is the matrix holding $\left\{V_{\mathrm{gaze}}^{i}\right\}$ in the whole training set. In combination with the loss function given as formula (15), the value of β is iterated and optimized with the Levenberg–Marquardt method [25].

### 2.5. Sampling and Denoising of Calibration Points

#### 2.5.1. Sampling Strategy of Calibration Points

To fit the regression model, some calibration points are sampled to build the training set. When the user gazes at each calibration point, the eye images and the coordinates of the gaze points are collected. They can be transformed to the pairs of input feature $V_{\mathrm{pc}}$ and output feature $V_{\mathrm{gaze}}$. To prevent appreciable extrapolation errors and ensure the stable performance of HMGT, the calibration area should be determined by human’s field of view. In particular, for the horizontal field of view of a human, symbol recognition and 3D perception happen within 60° of the central field of view, and for the vertical field of view of human, the optimum eye rotation degrees range from −30°∼25° [26]. Thus, the vector of visual axis is determined by eyeball horizontal rotation angle α, and vertical rotation angle β, where α ∈−30°∼30°, β∈−30°∼25°. Assuming that the origin of the eyeball coordinate system is the eyeball center, the *Z*-axis points to the horizontally forward direction, the *Y*-axis points to the vertically upward direction. The vector of visual axis can be defined by

**Figure 5:**
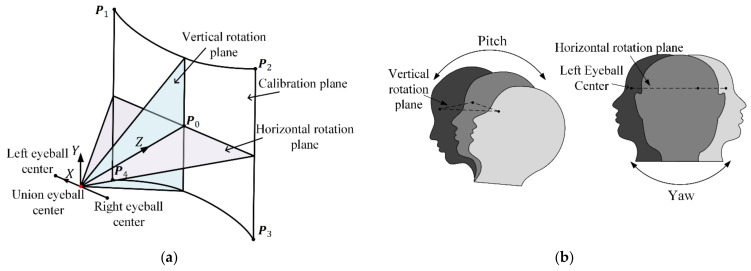
Illustration of sampling strategy for calibration points. (**a**) The sampling range on calibration plane has four vertexes $P_{1},P_{2},P_{3},P_{4}$ as limits. The polar coordinate $\left(\alpha ,\beta \right)$ of $P_{0}$, $P_{1},P_{2},P_{3},P_{4}$ are $\left(0{}^{\circ},0{}^{\circ}\right)$, $\left(-30{}^{\circ},25{}^{\circ}\right)$, $\;\left(30{}^{\circ},25{}^{\circ}\right)$, $\left(30{}^{\circ},-30{}^{\circ}\right)$, $\;\left(-30{}^{\circ},-30{}^{\circ}\right),\;\mathrm{respectively}$. (**b**) Calibration of the eyeball coordinate system. The left eye is taken as an example. The rotation pattern yaw and pitch are used to create horizontal rotation plane (X−Z plane) and vertical rotation plane (Y−Z plane).

(17) V ebgaze α,β=RyαRxβV0

where $V_{0}$ is the unit direction vector of *Z*-axis. $V_{0}={\left[0,0,1\right]}^{T}$, $R_{x}\left(\beta \right)$ and $R_{y}\left(\alpha \right)$ denote rotation matrices around *X*-axis and *Y*-axis, respectively, and V ebgaze  denotes the vector of visual axis in the eyeball coordinate system. Considering that the uniformity of training set has an influence on model fitting, the values of α and β should be uniformly distributed over their value range. To simplify the calibration procedures, the calibration points of both eyes are sampled together by defining the union eyeball coordinate system, as shown in Figure 5a. The midpoint of the left and right eyeball center is defined as the origin of the union eyeball coordinate system. The sampling points on calibration plane are calculated by intersecting the predefined visual axes and the plane.

Obviously, the pose of the eyeball coordinate system needs to be estimated for calibration point sampling. As shown in Figure 5b, the user is required to rotate the head in two different patterns (pitch and yaw) while looking straight ahead. When the user rotates the head, the coordinate of the eyeball center in the world coordinate system can be calculated by

(18) E world1=Ttra0world·E tra01

where Ttra0world is the transformation matrix between the Tracker-0 and world coordinate system which can be obtained in real time. E tra0 is the coordinate of eyeball center in the Tracker-0 coordinate system. Multiple values of E world collected in pattern pitch can be used to create the horizontal rotation plane of eyeball, multiple values of E world collected in pattern yaw can be used to create the vertical rotation plane of eyeball. In this way, the rotation matrix Rebworld between the eyeball and the world coordinate system can be estimated. Additionally, the rotation matrix Rebsc between the eyeball and the scene camera coordinate system can be calculated by

(19) Rebsc=Rtra0world−1·Rebworld·Rsctra0−1

where Rtra0world is the collected rotation matrix between the Tracker-0 and world coordinate system when the user gazes straight ahead. The transformation matrix between the eyeball and scene camera coordinate system can be represented as

(20) Tebsc=RebscE sc01

where E sc is calculated in Section 2.4. The eyeball coordinate system calculated by this method is not accurate, but it is still acceptable because sampling the calibration points over the rough field of view is enough for preventing appreciable extrapolation errors. The estimated rotation matrix Rebsc for both eyes is the same, thus the union eyeball coordinate system is calculated as

(21) Tu−ebsc=RebscE scu01

where E scu=E scleft+E scright2,  Tu−ebsc represents the transformation between the union eyeball coordinate system and the scene camera coordinate system, which is employed for calibration point sampling.

#### 2.5.2. Denoising Strategy of Calibration Points

In calibration point sampling, the 2D pupil center is extracted from eye image when the user gazes at each calibration point. However, the coordinates of the 2D pupil center may fluctuate because of the noise of the image and algorithm, especially when the pupil contour is partially obscured by the eyelid, which may lead to appreciable error of pupil center detection. In addition, the user may get distracted and not gaze calibration points, which results in the collection of outliers. Consequently, it is significant to denoise during data collection and remove outliers after data collection.

- Denoising in Data Collection

For each calibration point, *n* eye image frames are sampled and processed to get the 2D pupil center, respectively. The set of pupil centers are denoted as $\Omega =\left\{p_{i}\right\},i=1,2,\cdots ,n.$ The aggregation property of samples is used to denoise. The valid set is defined as

(22) $$\Omega_{\mathrm{valid}}=\left\{p_{i}\left|\left\|p_{i}-p_{\mathrm{median}}\right\|\right.<r_{\mathrm{noise}}\right\}$$

where $p_{\mathrm{median}}$ is the median value of the pupil centers’ coordinates in set Ω,$\;r_{\mathrm{noise}}$ is the pupil centers’ noise radius, whose value is set empirically. The number of coordinates in set $\Omega_{\mathrm{valid}}$ is $n_{\mathrm{valid}}$. When the proportion calculated by $\frac{n_{\mathrm{valid}}}{n}$ is too small (e.g.,$\;\frac{n_{\mathrm{valid}}}{n}<0.5$), collected data for this calibration point would be discarded, otherwise $p_{\mathrm{median}}$ is regarded as the 2D pupil center for current calibration points.

- Removing Outliers after Data Collection

Assuming that N calibration points are sampled, the collected data can be processed to a set $ℵ=\left\{\left(V_{\mathrm{pc}}^{i},V_{\mathrm{gaze}}^{i}\right)\right\}$, where i=1,2,⋯,N; The set ℵ is utilized to fit the regression model described in Section 2.4, the angular error of visual axis for the k’th data is calculated as

(23) $$err_{k}=\arccos\left(\frac{\beta_{ℵ}\cdot V_{\mathrm{pc}}^{k}\cdot V_{\mathrm{gaze}}^{k}}{\left\|\beta_{ℵ}\cdot V_{\mathrm{pc}}^{k}\|\|V_{\mathrm{gaze}}^{k}\right\|}\right)$$

where $\beta_{ℵ}$ is the calculated regression parameters with the set ℵ. The value of $err_{k}$ would be relatively large if the k’th data are an outlier, thus the k’th data are regarded as an inlier when $err_{k}<\tau$, where τ is an acceptable error.

### 2.6. Recalibration Strategy

In practical application scenarios, the slippage between HMGT and head would inevitably occur. In this situation, the calibrated parameters in the gaze estimation model are inapplicable, and recalibration is needed for the system to recover gaze estimation performance. However, it is undoubtedly a burden for users to carry out recalibration procedures that are as complex as the primary calibration. Therefore, it is essential to design an easy and efficient recalibration method.

When the slippage occurs, the new eyeball center E scnew and new rotation matrix Rebscnew between the eyeball and scene camera coordinate system can be estimated conveniently with the developed calibration tools as described in previous sections. In the new state, when a pair of data is collected and converted to input vector $V_{\mathrm{pc}}^{\mathrm{new}}\;\mathrm{and}\;$output vector $V_{\mathrm{gaze}}^{\mathrm{new}}$, Rebscnew and Rebsc can be used to switch them from the scene camera coordinate system in the new state (after slippage) to the scene camera coordinate system in the old state (before slippage). The calculation is as follows:

(24) Vgazeold=β0·ψVpcoldVpcold=Rebsc·Rebscnew−1·VpcnewVgazeold=Rebsc·Rebscnew−1·Vgazenew

where $\beta_{0}$ is the calibrated regression parameter. However, formula (24) is not rigorous. Firstly, the estimated rotation matrix Rebsc and Rebscnew are not precise as mentioned in Section 2.5, which means the calculated $V_{\mathrm{pc}}^{\mathrm{old}}$ and $V_{\mathrm{gaze}}^{\mathrm{old}}$ are not accurate. Secondly, the slippage results in the change in relative position between the eye camera and eyeball, which means the $V_{\mathrm{pc}}$ for the new state (after slippage) and old state (before slippage) are different, even if they are switched to the same reference coordinate system. Therefore, the calculated $V_{\mathrm{pc}}^{\mathrm{old}}$ is different from the ground-truth $V_{\mathrm{pc}}$ of the old state, and $\beta_{0}$ should be rectified. As a solution, a rotation vector $V_{r}$ is introduced to compensate for the orientation deviation, and a new regression parameter $\beta_{1}$ is employed. Assuming $V_{r}={\left[r_{1},r_{2},r_{3}\right]}^{T}$, the unit vector of $V_{r}$, $r={\left[\frac{r_{1}}{\left\|V_{r}\right\|},\frac{r_{2}}{\left\|V_{r}\right\|},\frac{r_{3}}{\left\|V_{r}\right\|}\right]}^{T}$, the rotation angle $\theta =\|V_{r}\|$, the modified formula is as follows,

(25) Vgazeold=β1·ψVpcoldVpcold=Rerror·Rebsc·Rebscnew−1·VpcnewVgazeold=Rerror·Rebsc·Rebscnew−1·VgazenewRerror=cosθΙ+1−cosθrrT+sinθr∧

where $R_{\mathrm{error}}$ denotes the rotation matrix that is converted from the rotation vector $V_{r}$ and $r^{\wedge }$ denotes the antisymmetric matrix of r. $\beta_{1}$ denotes the new regression parameter. Based on the formula (15) and Levenberg–Marquardt iteration method [25], $V_{r}$ and $\beta_{1}$ are iterated as unknown variables to find the optimal solution. As the orientation deviation caused by Rebsc and Rebscnew is small, three components of $V_{r}$ can be initialized as a small value such as$\;{\left[0.01,0.01,0.01\right]}^{T}$. The change in relative position between the eye camera and eyeball caused by slippage is small, so $\beta_{1}$ can be initialized as $\beta_{0}$. Considering that $V_{r}$ and $\beta_{1}$ both have initial values which are close to the optimal solution, recalibration does not need many calibration points in different gaze directions such as primary calibration, but only one or several calibration points for parameter iteration.

## 3. Experiment and Results

To verify the effectiveness of our proposed method, the HMGT shown in Figure 2 is developed. This HMGT has two eye cameras (30 fps, 1280 × 720 pixels) to capture movement of eyes, two scene cameras (30 fps, 1280 × 720 pixels) to capture scene view and a 6D pose tracker (Tracker-0) to capture the head movement. Before the experiment, the intrinsic matrix parameters of eye cameras and scene cameras are calibrated by the MATLAB toolbox. The transformation matrix between eye cameras, scene cameras and Tracker-0 is estimated by the proposed method in Section 2.2. Five subjects participate in the experiment. Firstly, the subject needs to calibrate the eyeball coordinate system with the proposed method in Section 2.3 and Section 2.5. Then, the calibration points and test points for regression model fitting are sampled in union eyeball coordinate system. In order to evaluate the effects of calibration depth on gaze estimation performance and compare different methods, calibration points at three different planes distant from the eyeball center with 0.3 m, 0.4 m and 0.5 m are taken into consideration. At each depth, 42 calibration points and 30 test points are sampled with uniform angular intervals of visual axis. The positions of them are calculated by intersecting the pre-defined visual axes and the calibration plane.

**Figure 6:**
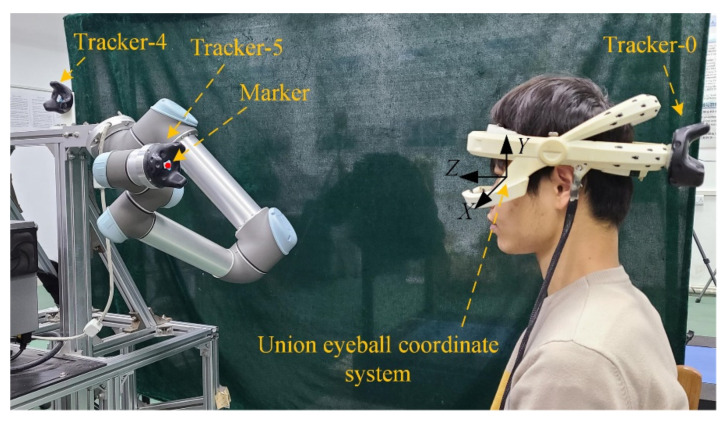
Calibration setup utilizing a UR robot arm and 6D pose trackers.

As shown in Figure 6, two 6D pose trackers, Tracker-4 and Tracker-5, are fixed with the arm base and the end effector, respectively. The robot arm can move the end-effector with a marker to a predefined location in the eyeball coordinate system with real-time head pose tracking. The 2D pupil center in eye image is detected in real time by the algorithm investigated in [27]. When the subject gazes at the marker, the 2D pupil center and the position of the marker are collected in synchronization. The data collection is implemented by using programming in C++. To verify the effectiveness of recalibration method, all subjects wear the HMGT twice and repeat the entire calibration twice. The gaze estimation model is implemented by using programming in MATLAB with collected data. Data acquired in the first wearing are used to evaluate the gaze accuracy of primary calibration method, and data acquired in the second wearing are used to evaluate the gaze accuracy of the recalibration method and compare different methods. The common criterion for evaluating gaze estimation performance is the angular error between estimated visual axis and real visual axis. However, it is improper to compare the performances of different methods with the angular error of visual axis derived with the estimated eyeball center and the gaze point, considering that the estimated eyeball centers in different methods usually have different error distributions. Therefore, a more reasonable evaluation criterion, scene angular error (*SAE*), is defined as

(26) $$SAE=\arccos\frac{V_{s}\cdot V_{e}}{\left\|V_{s}\|\cdot \|V_{e}\right\|}$$

where $V_{s}$ is the direction vector from the scene camera optical center to the real gaze point and $V_{e}$ is the direction vector from the scene camera optical center to the estimated gaze point. The estimated gaze point is the intersection of the estimated visual axes of two eyes.

### 3.1. Evaluation of Primary Calibration Method

**Figure 7:**
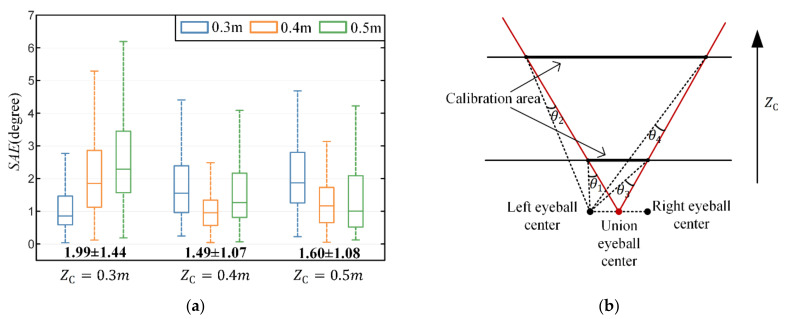
Gaze estimation performance of the primary calibration method. (**a**) Gaze estimation performance based on training set at different depths.$\;Z_{c}$ denotes the depth of the calibration plane. The bold black numbers at the bottom are the angular error in degrees (mean ± standard deviation). (**b**) The parallax between the single (left) and union eye visual system. As the depth of calibration plane increases, the parallax becomes smaller (e.g., $\theta_{1}>\theta_{2}$, $\theta_{3}>\theta_{4}$ ).

The gaze estimation performance of the primary calibration method based on a training set at different depths is shown in Figure 7a. It can be found that each situation achieves better performance than other situations at corresponding calibration plane. For example, the method achieves the best gaze estimation performance at a depth of 0.3 m when $Z_{C}=0.3\;m$. In addition, the mean and standard deviation of error in situation 1 ($Z_{C}=0.3\;m$) are significantly high while there is no significant difference between situation 2 ($Z_{C}=0.4\;m$) and situation 3 ($Z_{C}=0.5\;m$) (paired-t test: tstat=−1.56, p=0.1213). This may be caused by the extrapolation error. Because of the use of the union eyeball coordinate system, the field of view covered by calibration points at different depths is slightly different due to the depth-dependent parallax between the single and the union eye visual system (see Figure 7b). As the depth of calibration plane increases, the parallax becomes smaller, the difference in gaze estimation performance in different situations becomes smaller.

### 3.2. Evaluation of Recalibration Method

**Figure 8:**
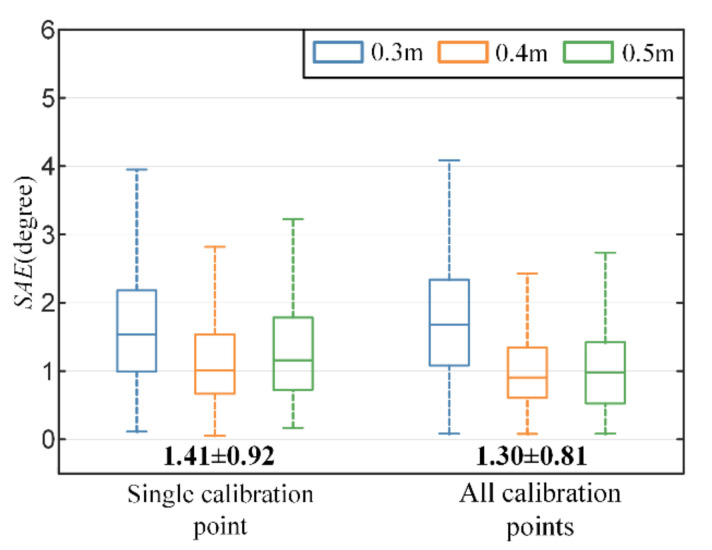
Comparison of primary calibration method and recalibration method with different number of calibration points. The bold numbers at the bottom are the angular error in degrees (mean ± standard deviation).

The proposed recalibration method re-estimates the transformation matrix between the eyeball and scene camera coordinate system with the proposed geometry-based method and utilizes calibration points to rectify the parameters of the gaze estimation model. To reveal the influence of the number of calibration points on gaze estimation performance in recalibration, two strategies are implemented and compared. One uses a single calibration point, and the other uses all calibration points at depth of 0.5 m. As mentioned in Section 2.5, the positions of calibration points on calibration plane are determined by eyeball horizontal rotation angle α, and vertical rotation angle β. Without loss of generality, the calibration point whose polar coordinate $\left(\alpha ,\beta \right)$ is closest to (0,0) is selected to verify the single-point strategy. As shown in Figure 8, the mean and standard deviation of error in situation 1 (single calibration point) are slightly higher than the other two situations. The overall gaze accuracy performance of them is comparable (the paired-t test: tstat=1.94, p=0.053).

### 3.3. Comparison with Other Methods

To compare our proposed method with other methods, we implemented and evaluated the following baseline methods.

- Nonlinear optimization

The method in [9] formulated a constrained nonlinear optimization to calculate the eyeball center and the regression parameters that were used to map the eye image features to the gaze vector. The initial position of the eyeball center is assumed by 2D pupil center and scene camera intrinsic matrix. The constrained search range of the eyeball center is set as $\pm \left[0.05\;m,\;0.05\;m,\;0.02\;m\right]$. This method needs two calibration planes, and the training set at depth of 0.3 m and 0.5 m is used for calculating.

- Two mapping surfaces

The method based on mapping surfaces [19] mapped the eye image feature to 3D gaze point on a certain plane. This way, two calibration surfaces with different depths correspond to two different regression mapping functions. For a particular eye image, two different 3D gaze points on different planes can be calculated, then the visual axis can be obtained by connecting two points. This method also needs two calibration planes and the training set at a depth of 0.3 m and 0.5 m is used for calculation.

**Figure 9:**
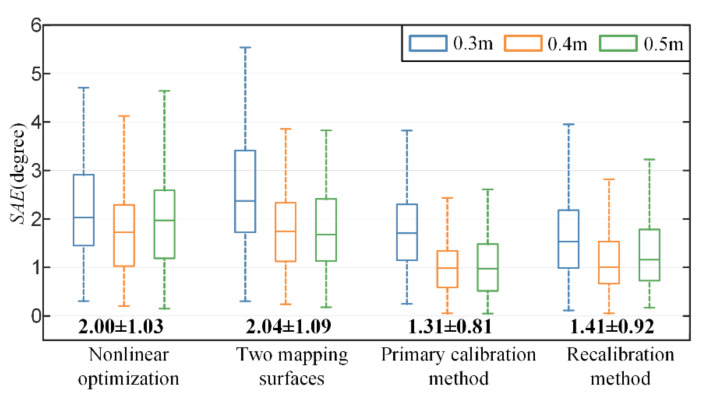
Comparison of the proposed method and state-of-the-art methods at different depths. The bold numbers at the bottom are the angular error in degrees (mean ± standard deviation).

In comparison, our proposed primary recalibration method and recalibration method use the training set at a depth of 0.5 m. As shown in Figure 9, the proposed primary calibration method achieves the lowest mean error, followed by the proposed recalibration method. There is no significant difference between their overall gaze estimation performance (the paired-t test: tstat=1.83, p=0.068). In addition, the mean error of the method with nonlinear optimization is slightly lower than the error of the method with two mapping surfaces. Compared to the method with nonlinear optimization, the proposed primary calibration and recalibration method improve accuracy by 35 percent (from a mean error of 2.00 degrees to 1.31 degrees) and 30 percent (from a mean error of 2.00 degrees to 1.41 degrees).

**Figure 10:**
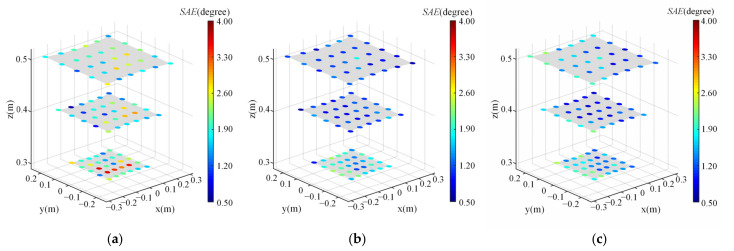
The scene angular error at each of the 90 test points for different methods. Noted that the error of each test point is calculated by averaging the error of the same test point for all subjects. The coordinates of these test points are represented in union eyeball coordinate system. (**a**) Nonlinear optimization. (**b**) Primary calibration method. (**c**) Recalibration method.

The scene angular error at each of the 90 test points for different methods is illustrated in Figure 10. The error of each test point is calculated by averaging the error of the same test point for all subjects. The primary calibration and recalibration method obtain better accuracy performance than the baseline method for the 81% of validation points. Although the accuracy performance of our proposed method at a few points is worse than the baseline method, the error at these points is relatively low (lower than 2.4 degree) which is acceptable. In terms of time cost, the baseline method cost 168 s on average while the proposed primary calibration and recalibration method cost 114 s and 32 s, respectively. Therefore, it can be concluded that the proposed methods can achieve better accuracy performance with less time cost of calibration procedures.

## 4. Discussion

As revealed by the comparison of different methods, the proposed gaze estimation method achieves better performance than the state-of-the-art methods. The main reason is that the eyeball and camera coordinate system are estimated accurately in advance so that they are used as known knowledge to simplify the mapping relationship in regression model. When slippage occurs, the proposed recalibration strategy can utilize the old regression parameters as initial value to optimize the new regression parameters with estimated eyeball coordinate system. That is why the recalibration can get comparable performance with primary calibration with a single calibration point. As a limitation, our proposed calibration and recalibration method both require the calibration procedure to estimate the transformation matrix between eyeball and scene camera coordinate system, but it is simple and it takes little time (30 s approximately).

To compare our proposed method with other methods which need multiple calibration depths, the robot arm is adopted in our experiments to sample calibration points at different depths. However, our proposed method has no requirement for multiple calibration depth, thus the robot arm is not necessary for practical use. For instance, the combination of display screen and trackers can be adopted to sample calibration points at a certain depth, which is more convenient. Noted that the use of the 6D pose tracker can help adjust the positions of calibration points with the movement of a human’s head. It is user friendly because there is no need to keep the head still when sampling calibration points. Benefits always come with costs. The main disadvantage of our proposed method is that the 6D pose tracker is necessary for calibration procedures. However, the head pose tracking based on the 6D pose tracker is beneficial for human–machine interaction because the estimated visual axis can be switched to the world coordinate system.

## 5. Conclusions

In this article, we propose a high-accuracy hybrid 3D gaze estimation model for HMGT with head pose tracking. The two key parameters, eyeball center and camera optical center, are accurately estimated in the head frame with a geometry-based method, so that a low-complexity mapping relationship between two direction features can be established with a quadratic polynomial model. The input feature is the unit direction vector from the eye camera optical center to virtual pupil center and the output feature is the unit direction vector of visual axis. The direction features for model fitting are sampled with uniform angular intervals over human’s field of view, which can help to acquire a high-quality training set and prevent appreciable extrapolation error. For the slippage between HMGT and the head, an efficient recalibration method is proposed with single calibration point after recalculating the eyeball coordinate system. The experiment results indicate that both the primary calibration method and recalibration method achieve higher gaze accuracy than state-of-the-art methods. Generally, the advantages of the proposed method are increasing the gaze estimation accuracy, improving the calibration point sampling strategy and reducing the burden of calibration procedures. The disadvantage is that the 6D pose tracker is necessary for calibration procedures. In future work, the robustness of the proposed gaze estimation model should be discussed and improved.

## Data Availability

The data that support the findings of this study are available from the corresponding author upon reasonable request.
